# Three-Dimensional Virtual Reconstructions of Shoulder Movements Using Computed Tomography Images: Model Development

**DOI:** 10.2196/48381

**Published:** 2023-10-05

**Authors:** Yu-Hee Kim, In Park, Soo Buem Cho, Seoyon Yang, Il Kim, Kyong-Ha Lee, Kwangnam Choi, Seung-Ho Han

**Affiliations:** 1 Advanced Biomedical Research Institute Ewha Womans University Seoul Hospital Seoul Republic of Korea; 2 Department of Orthopedic Surgery Ewha Womans University College of Medicine Seoul Republic of Korea; 3 Department of Radiology Ewha Womans University College of Medicine Seoul Republic of Korea; 4 Department of Rehabilitation Medicine Ewha Womans University College of Medicine Seoul Republic of Korea; 5 SurgicalMind Inc Gwangju Republic of Korea; 6 Division of National S&T Data Korea Institute of Science and Technology Information Daejeon Republic of Korea; 7 Department of Anatomy Ewha Womans University College of Medicine Seoul Republic of Korea; 8 Ewha Medical Academy Ewha Womans University Medical Center Seoul Republic of Korea

**Keywords:** human digital twin, musculoskeletal twin, shoulder movement, visualization application, digital twin, musculoskeletal, visualization, movement, joint, shoulder, tomography, development, animation, animated, anatomy, anatomical, digital health, representation, simulation, virtual

## Introduction

The demand for digital reconstruction of the human body, the “human digital twin,” is increasing. Areas where human digital twins can be used include supporting disease diagnoses and predicting various treatment outcomes [1,2]. However, accurate digitalization of the structure and function of the human body is required, and a digital twin of the whole skeleton will provide a broader understanding of the human body. The Digital Korean Project, which was conducted from 2003 to 2007, established a database of Korean skeletons that included physical properties [3] and is being used in various research fields [4,5]. Digital representations of the human skeleton can be constructed at a higher level by including movement. Recently, Hernigou et al [6] proposed a method to include an ankle motion axis in digital twins. Although this method can be useful, one drawback of this study was that muscle movement was not included. This is because human skeletal movements cannot be evaluated except for the muscles. Therefore, in this study, we aimed to develop a visualization application of human shoulder movements involving bones and muscles.

## Methods

### Overview

Computed tomography (CT) images of the shoulders obtained at Ewha Womans University Seoul Hospital were used for 3D modeling. CT images in the DICOM file format were converted to an STL file format and then imported into the Blender program (Blender Institute). The size and volume of each bone and muscle involved in shoulder movement were measured and used for image visualization and 3D modeling. For the virtual reconstruction of shoulder movement, the Controller function was used to crudely model bone and muscle movement, and the Shapekey function was used to further adjust muscle contraction, relaxation, and rotation.

The Unity engine (Unity Technologies, San Francisco, CA) was used to develop a real-time 3D visualization application of shoulder movements. Reconstructed 3D resources and shoulder movement animations were exported from the Blender program in the FilmBoX file format and imported as Unity assets. A separate collision box was generated to avoid slowdown during the real-time selection of bones and muscles in polygonal units. Unity cameras were customized to display 3D structures and animations from different viewpoints. Interactive visibility controls for each bone and muscle were designated, and animation sequences were programmed for each motion.

### Ethical Considerations

This study received ethics approval from Ewha Womans University Seoul Hospital (ethics approval No. 2023-02-037).

## Results

**Figure 1:**
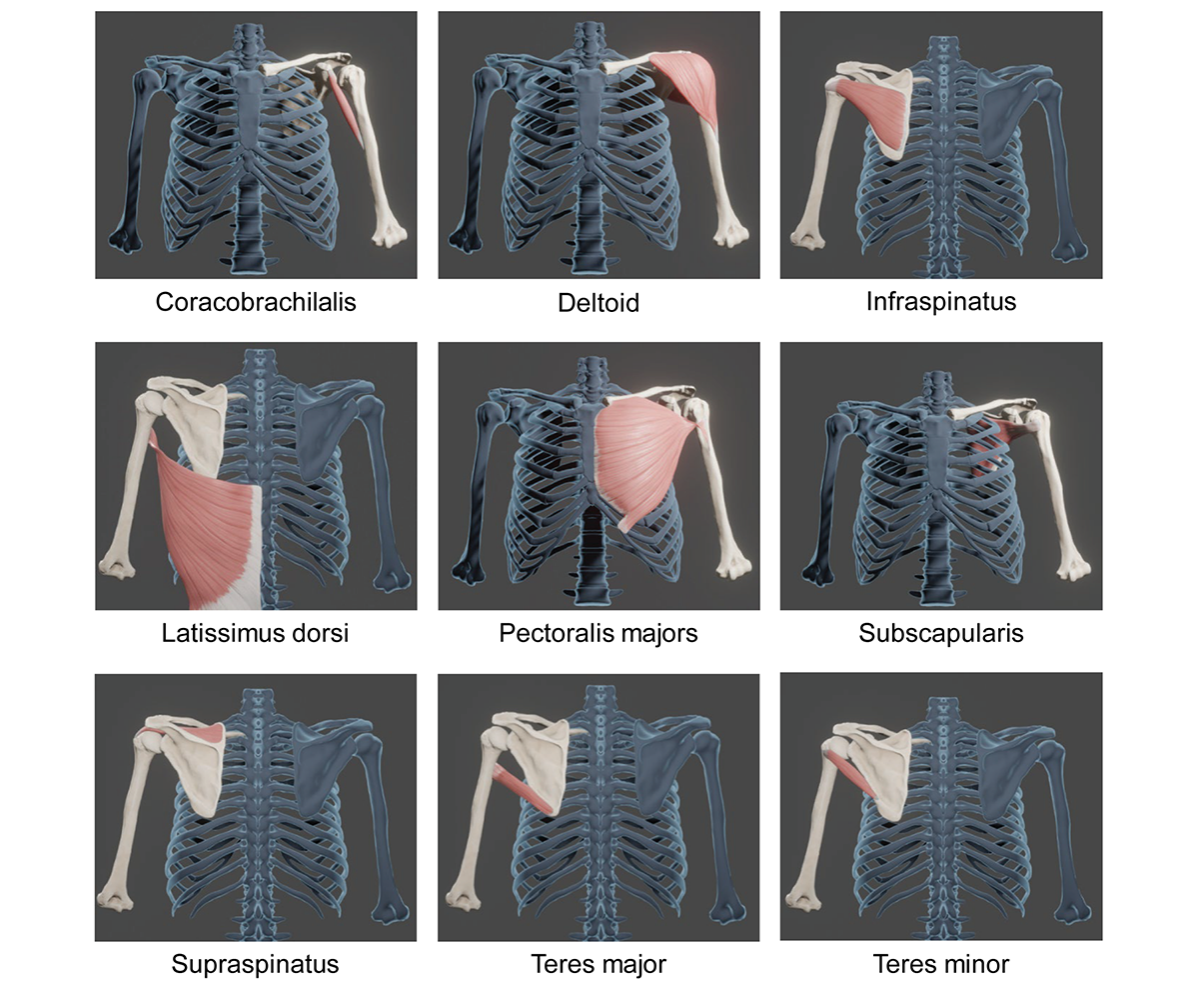
Three-dimensional reconstruction of muscles involved in shoulder movement. The muscles involved in shoulder movement, which were manually segmented using computed tomography DICOM files, were reconstructed in 3D and placed on the bones.

To build a 3D model of the shoulders, 3 bones (scapula, clavicle, and humerus) were constructed to accommodate 6 shoulder movements (flexion, extension, abduction, adduction, internal rotation, and external rotation), and then, 9 muscles (coracobrachialis, deltoid, infraspinatus, latissimus dorsi, pectoralis majors, subscapularis, supraspinatus, teres major, and teres minor) that are primarily involved in each movement were built [7]. The reconstructed bones and muscles have been anatomically validated and are shown in Figure 1. These were then used to construct animations of muscle movements (Multimedia Appendix 1). The alignment and rotation angles of bones in the normal range of shoulder movement were implemented in the animation based on a kinesiology textbook [8].

**Figure 2:**
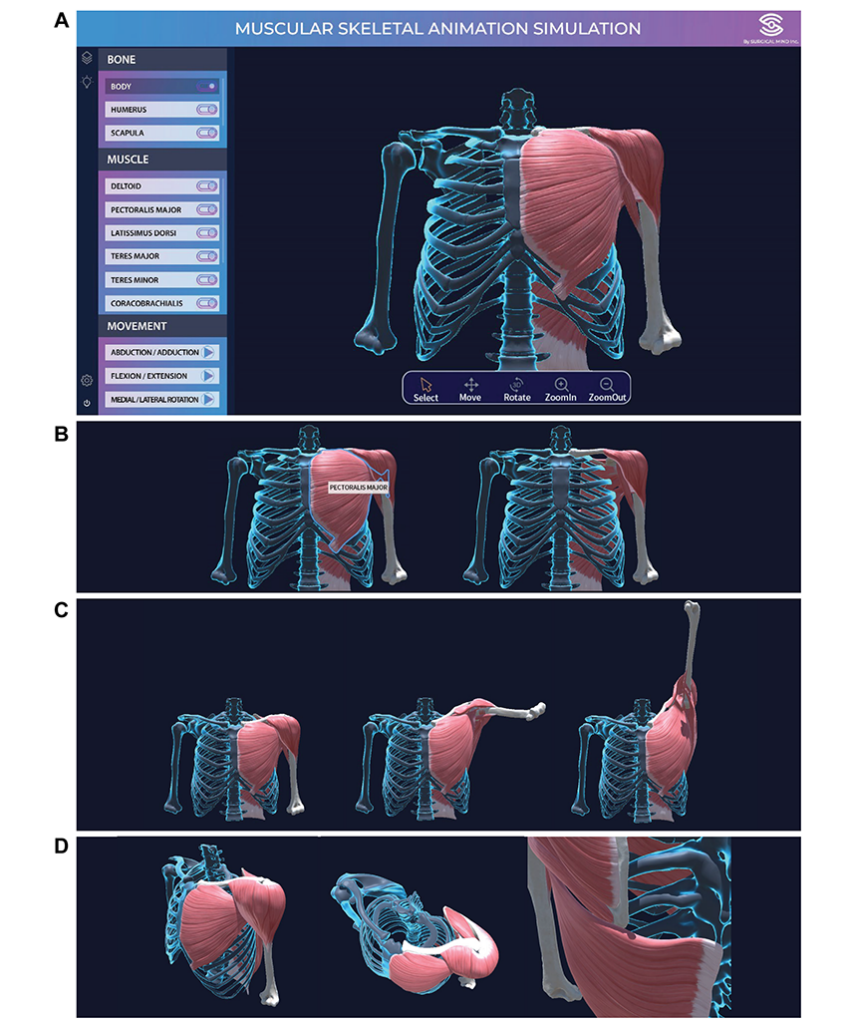
Application for visualization of shoulder movement. (A) A visualization application for real-time simulation of shoulder movement was constructed. Functions included in the application are shown. (B) Present the structure name and change the visibility; (C) play the movement animation; and (D) move, rotate, or zoom in/out of the structure.

Next, we developed a visualization application using the digital reconstruction of shoulder movements. The user interface of the application is shown in Figure 2A. The following functions were programmed in the application: present the name of the structure where the mouse is located; change the visibility of bones and muscles (Figure 2B); play/stop shoulder movement animation through the animation bar (Figure 2C); and move, rotate, or zoom in/out of the entire structure (Figure 2D). Using this application, we demonstrated a 3D real-time visualization of each structure and movement of interest.

## Discussion

The ultimate goal of a human digital twin is the development of a comprehensive whole-body digital twin that includes the body structure and movement. This will enable the modeling of various diseases at the systemic level without limitation to a single organ or stationary postures. In this study, we used CT images to build a 3D digital representation of the shoulders comprising bones and muscles, and developed an application for visualizing shoulder movements. To the best of our knowledge, this is the first report implementing an application for the visualization of shoulder movements involving bones and agonistic muscles.

Future research will lead to the development of an algorithm to import patient-specific CT data and present it in our 3D visualization application. This includes an automated system that can identify normal and abnormal ranges of motion and can be used for the development of medical applications in arthroplasty or rehabilitation. Furthermore, it will be possible to develop an evolving digital twin that can continuously reflect and update changes that occur during the human life cycle.

## Appendix

Multimedia Appendix 1 Animation of muscle contraction, relaxation, and rotation.

## Abbreviations

CT: computed tomography
